# Papillary Thyroid Cancer and Lung Adenocarcinoma Presenting as Two Primary Malignancies in a Patient with Symptomatic Goiter

**DOI:** 10.1155/2015/394958

**Published:** 2015-07-28

**Authors:** Deepu Daniel, Leah Delumpa, Natasha Bray

**Affiliations:** Broward Health Medical Center, Fort Lauderdale, FL 33316, USA

## Abstract

In rare instances, patients may be diagnosed with two different primary malignancies. Though such synchronous malignancies have been documented in sporadic case reports, the overwhelming majority of malignancies involving multiple organs can be attributed to a primary source. Papillary thyroid carcinoma and lung adenocarcinoma are rarely diagnosed within the same year. Our case report presents a patient who was diagnosed with these two malignancies during her same hospital visit. Biopsies results proved that the two malignancies were in fact separate entities and not a consequence of metastasis from a primary source.

## 1. Introduction

Papillary thyroid carcinoma is the most frequently diagnosed form of thyroid cancer, constituting up to 80% of cases [1, 2]. Females are affected more than males by a ratio of 3 : 1 [3]. Historically, thyroid carcinoma has been associated with head and neck radiation treatment before 1960. However, there is still an increasing incidence of thyroid carcinoma today despite the decline of radiation treatment for benign head and neck conditions. This is thought to be secondary to earlier screening which has caused 10-year survival rates to increase to 95% [3].

Papillary carcinoma usually presents as a slow growing neck nodule found incidentally on physical exam or imaging. Physical findings associated with malignancy include tracheal compression in the absence of a goiter, a fixed, hard nodule increasing in size, cervical lymphadenopathy, and rising levels of TSH. Many patients, however, present with nonspecific physical findings [4].

Papillary thyroid carcinoma metastasizes via the lymphatic system with an incidence as high as 64% [5]. Later in the disease course, it metastasizes hematogenously, most frequently to bone and lung [1]. Several studies have also shown patients with thyroid cancer to have an 11–30% risk of developing another malignancy when compared to the general population. Thyroid malignancies have been associated with other cancers such as breast, prostate, kidney, salivary, scrotal, neural, and leukemia [3, 6]. In this case report, we present a patient with synchronous papillary thyroid adenocarcinoma alongside lung adenocarcinoma with a concurrent paraneoplastic syndrome.

## 2. Case Presentation

A 67-year-old Hispanic female presented to the emergency room with a chief complaint of progressive weakness and increased neck girth for the past several months. She had been seen by her primary care physician one week prior to presentation where she was diagnosed with hypothyroidism and started on levothyroxine 75 mcg daily. Due to the progression of generalized weakness and neck swelling, the patient presented to the emergency department for further evaluation. In addition, the patient had also been experiencing mild episodes of dysphagia to solids, odynophagia, and decreased appetite but denied any significant weight loss at this time. Patient denied any past medical or surgical history other than previously mentioned. This patient was a nonsmoker and had no significant radiation therapy or exposure in the past.

**Figure 1:**
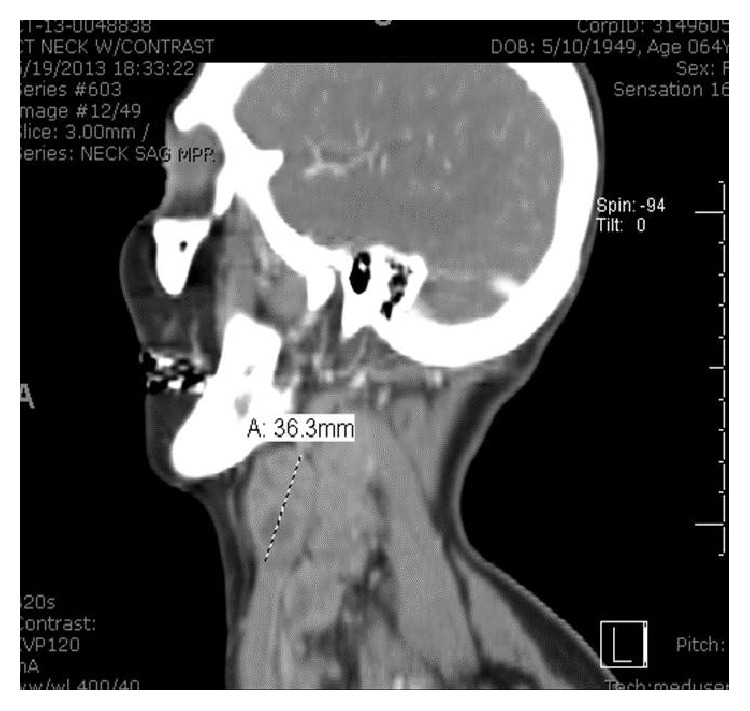
CT of the neck indicating the largest lymph node on left measuring 36.3 mm.

**Figure 2:**
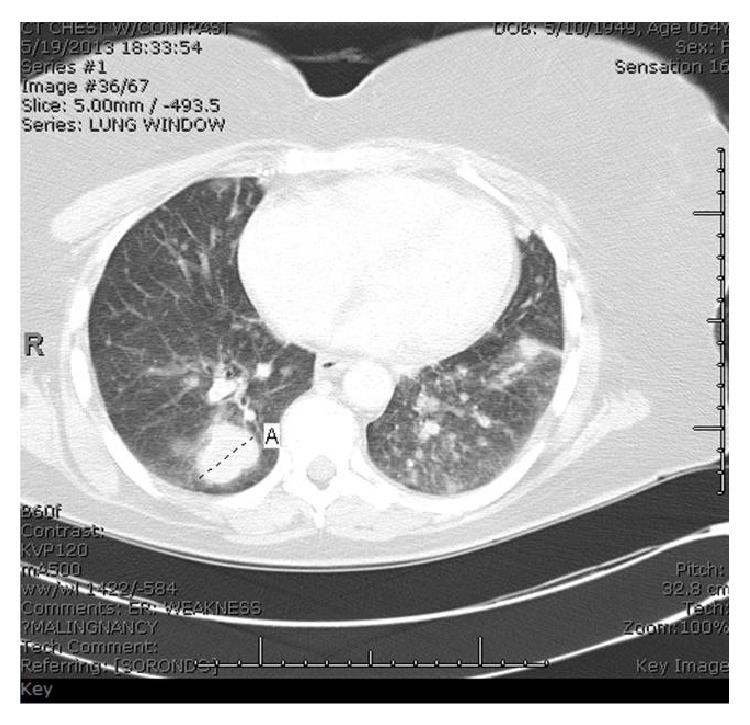
CT of the chest showing bilateral pulmonary nodules.

**Figure 3:**
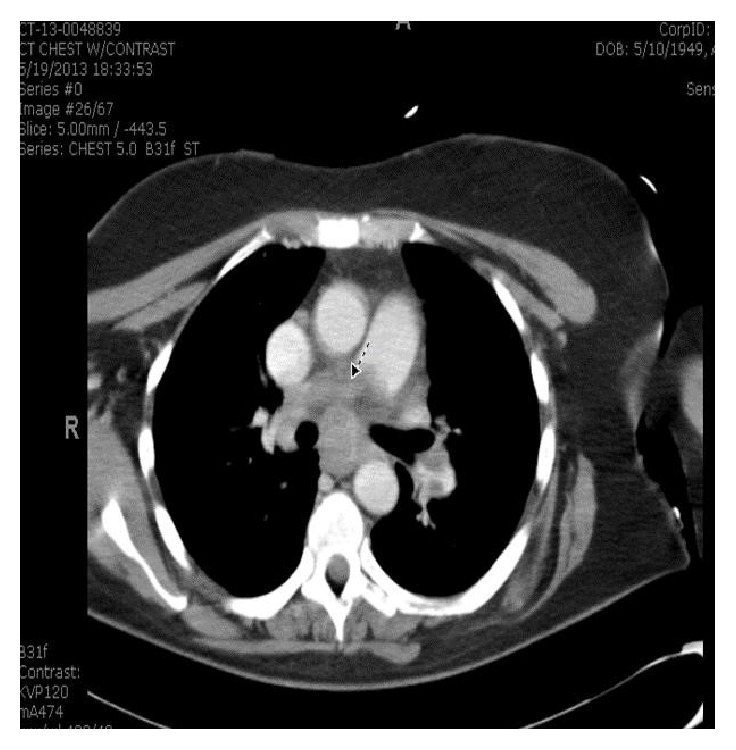
CT of the chest with arrow demarcating a saddle embolus.

Upon presentation, vital signs were stable. Significant physical exam findings included an enlarged thyroid gland with multiple enlarged lymph nodes in the posterior auricular regions. Pertinent lab studies included a white blood cell count of 13.3 × 10^3^/*μ*L, thyroid stimulating hormone level of 8.66 *μ*IU/mL, and a free thyroxine of 1.04 ng/dL. Imaging studies included a cat scan (CT) of the neck with contrast seen in Figure 1. This revealed necrotic lymphadenopathy and multiple thyroid nodules bilaterally. A previous thyroid ultrasound 2 months prior had indicated the presence of bilateral thyroid nodules which were described as solid and hypoechoic. CT of the chest with contrast, Figures 2 and 3, showed multiple bilateral pulmonary nodules and a saddle pulmonary embolism and multiple small hepatic masses and bilateral adrenal masses.

Patient was started on full dose of Lovenox for anticoagulation of the pulmonary embolism. Endocrinology and hematology/oncology were consulted for the multiple nodules in the thyroid, lung, liver, and adrenal glands.

During the hospital course, an ultrasound guided biopsy of the thyroid and a CT guided biopsy of the right lung nodule were obtained. The patient also developed a blanching erythematous maculopapular rash on the anterior chest, abdomen, back, and proximal upper thighs. Dermatology was consulted. Workup revealed the serological marker Anti-Jo was elevated at 236 units/mL.

**Figure 4:**
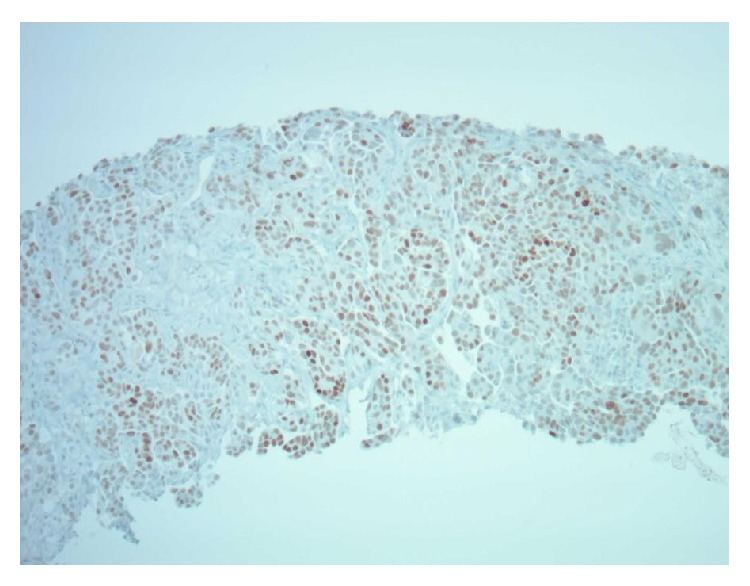
Thyroid biopsy: TTF1 immunostain 100x magnification in neck mass. Brown is positive in nucleus. Thyroid transcription factor-1 is a nuclear protein important in regulating gene expression in thyroid and lung tissue.

**Figure 5:**
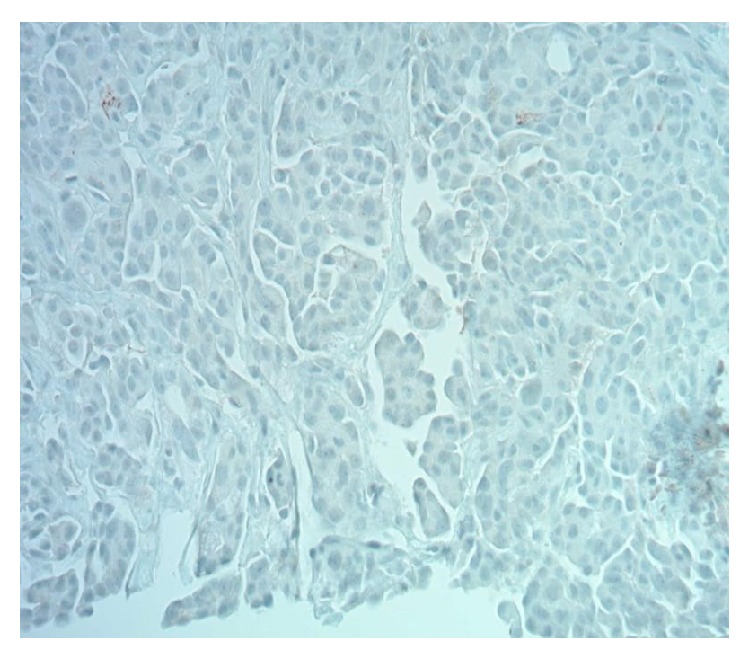
Thyroid biopsy: Napsin A immunostain 200x in neck mass. Napsin A is sensitive and specific for pulmonary adenocarcinoma. Tumor is negative.

**Figure 6:**
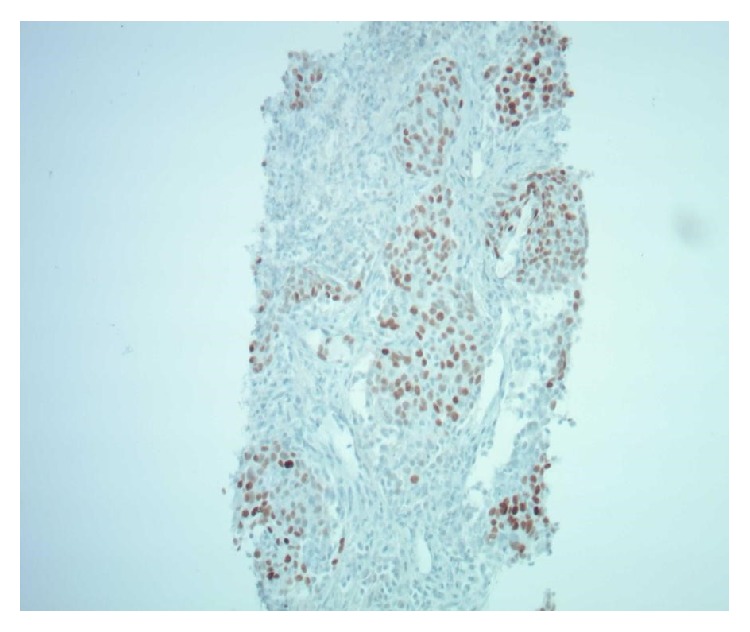
Lung biopsy: TTF1 immunostain in lung 100x magnification. Brown is positive in nucleus.

**Figure 7:**
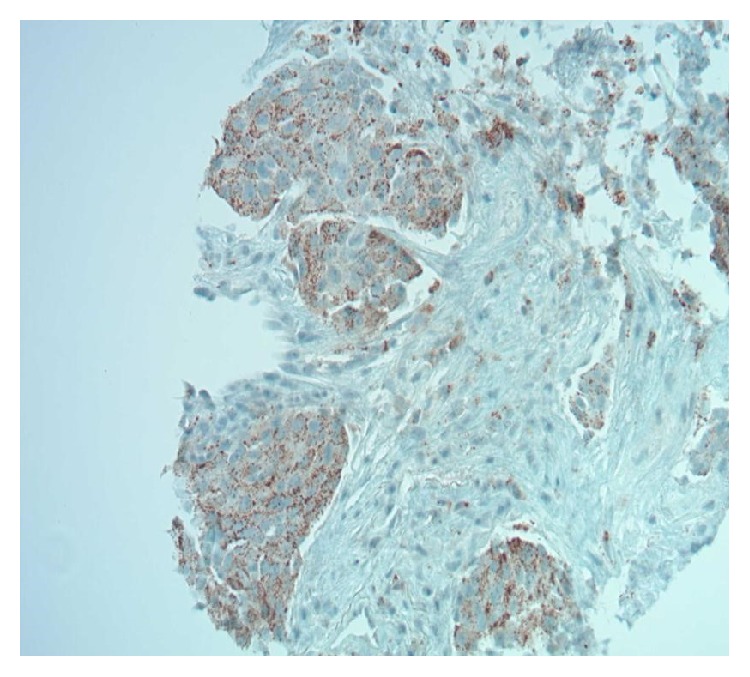
Lung biopsy: Napsin A immunostain 200x in lung is positive.

Core biopsy of the right thyroid nodule revealed adenocarcinoma with specimen containing papillae and psammoma bodies seen in Figures 4 and 5. Concurrently, the right lung biopsy was positive for adenocarcinoma. The morphology and the immunophenotype of the lung tissue were distinct from the thyroid biopsy (Figures 6 and 7).

These findings suggested the presence of two different primary malignancies. The patient's final diagnosis was papillary thyroid carcinoma and adenocarcinoma of the lung with metastasis to the adrenal glands and liver.

Palliative chemotherapy with cisplatin and altima was decided for treatment of the lung malignancy, and a port was placed before discharge. She was to receive at least 4 cycles of chemotherapy prior to a thyroidectomy at a later date. Unfortunately, the patient's functional status declined rapidly. The patient passed away a few months after the initial hospital admission.

## 3. Discussion

This patient was diagnosed with two distinct malignancies, papillary thyroid adenocarcinoma and lung adenocarcinoma. Synchronous malignancies are discovered within the same year as the first primary cancer. The second malignancy is usually discovered either incidentally during surgery, or during diagnostic workup for the first malignancy as was the case of this patient [7]. These patients are older at the time of diagnosis, present with a thyroid mass, and have more advanced staged disease which are characteristics of this patient [8].

The type of synchronous malignancy upon presentation was atypical for papillary thyroid adenocarcinoma along with the clinical course. The most common associated malignancy in women is breast occurring in 36% of second cancers [3, 7]. Literature search did not find a consensus on the incidence synchronous lung and thyroid cancer. Ronckers et al. had found decreased number of observed cases to expected cases of lung carcinoma with concurrent differentiated papillary thyroid carcinoma in the US surveillance, epidemiology, and end result database [3]. However, smaller single center studies such as the one by Ömür et al. had found that lung carcinoma was the second most common malignancy occurring in 3 of 15 cases observed synchronous thyroid carcinoma [7].

Lung adenocarcinomas are normally associated with paraneoplastic syndromes such as hypercalcemia from secretion of parathyroid hormone related protein and Cushing's syndrome from secretion of ACTH [9]. In the course of the hospital stay, the patient was found to be anti-jo positive. Anti-jo is an autoantibody that is specific to dermatomyositis, characterized by symmetric proximal muscle weakness and dermatologic manifestations. Dermatomyositis is diagnosed simultaneously with malignancies in one-third of patients [10]. It also has a high incidence of disease associated malignancy such as cervical, lung, ovarian, pancreatic, bladder, and stomach cancers [11]. During the hospital course patient's overall health rapidly deteriorated and eventually became fully dependent on family. This atypical paraneoplastic syndrome with synchronous metastatic cancers contributed to the poor prognosis of this patient.

Our patient lacked several risk factors for thyroid and lung malignancies. She denied any significant family history of malignancy, previous head and neck radiation, smoking history, and harmful environmental or work exposures. Therefore it is seemingly difficult to postulate a hypothesis for the occurrence of these two primary malignancies. A possibility is that a more extensive family history may have brought to light occurrences of cancer within her extended family. If so, this would suggest a genetic component as the cause for her two primary malignancies. Further genetic and molecular studies may have shown certain aberrancies that could have possibly led to her condition.
